# Regulation of *nc886 (vtRNA2-1)* RNAs is associated with cardiometabolic risk factors and diseases

**DOI:** 10.1186/s13148-025-01871-7

**Published:** 2025-04-29

**Authors:** Sonja Rajić, Thomas Delerue, Justiina Ronkainen, Ruiyuan Zhang, Joanna Ciantar, Daria Kostiniuk, Pashupati P. Mishra, Leo-Pekka Lyytikäinen, Nina Mononen, Laura Kananen, Annette Peters, Juliane Winkelmann, Marcus E. Kleber, Stefan Lorkowski, Mika Kähönen, Terho Lehtimäki, Olli Raitakari, Melanie Waldenberger, Christian Gieger, Winfried März, Emily W. Harville, Sylvain Sebert, Saara Marttila, Emma Raitoharju

**Affiliations:** 1 Molecular Epidemiology, Faculty of Medicine and Health Technology, Tampere University, Tampere, Finland; 2 Research Unit Molecular Epidemiology, Helmholtz Zentrum München, German Research Center for Environmental Health, Neuherberg, Germany; 3 Research Unit of Population Health, Faculty of Medicine, University of Oulu, Oulu, Finland; 4 Department of Epidemiology, School of Public Health and Tropical Medicine, Tulane University, New Orleans, LA USA; 5 Department of Clinical Chemistry, Faculty of Medicine and Health Technology, Tampere University, Tampere, Finland; 6 Finnish Cardiovascular Research Center Tampere, Faculty of Medicine and Health Technology, Tampere University, Tampere, Finland; 7 Fimlab Laboratories, Tampere, Finland; 8 Tampere University Hospital, Wellbeing Services County of Pirkanmaa, Tampere, Finland; 9 Department of Medical Epidemiology and Biostatistics, Karolinska Institutet, Stockholm, Sweden; 10 Faculty of Social Sciences (Health Sciences), Gerontology Research Center, Tampere University, Tampere, Finland; 11 Department of Neurobiology, Care Sciences and Society (NVS), Karolinska Institute, Stockholm, Sweden; 12 Institute of Epidemiology, Helmholtz Zentrum München, German Research Center for Environmental Health, Neuherberg, Germany; 13 Institute of Medical Information Sciences, Biometry and Epidemiology, Ludwig-Maximilians-University, Munich, Germany; 14 German Research Center for Cardiovascular Disease (DZHK), Partner Site Munich Heart Alliance, Munich, Germany; 15 Institute of Neurogenomics, Helmholtz Zentrum München, Munich, Germany; 16 Institute of Human Genetics, Technical University, Munich, Germany; 17 Munich Cluster for Systems Neurology (SyNergy), Munich, Germany; 18 Vth Department of Medicine (Nephrology, Hypertensiology, Endocrinology, Diabetology, Rheumatology), Medical Faculty of Mannheim, University of Heidelberg, Heidelberg, Germany; 19 SYNLAB MVZ Humangenetik Mannheim, Mannheim, Germany; 20 Institute of Nutritional Sciences, Friedrich-Schiller-University, Jena, Germany; 21 Competence Cluster for Nutrition and Cardiovascular Health (nutriCARD) Halle-Jena-Leipzig, Jena, Germany; 22 Department of Clinical Physiology, Tampere University Hospital and Faculty of Medicine and Health Technology, Tampere University, Tampere, Finland; 23 Centre for Population Health Research, University of Turku and Turku University Hospital, Turku, Finland; 24 Research Centre of Applied and Preventive Cardiovascular Medicine, University of Turku, Turku, Finland; 25 Department of Clinical Physiology and Nuclear Medicine, Turku University Hospital, Turku, Finland; 26 Synlab Academy, SYNLAB Holding Deutschland GmbH, Augsburg & Mannheim, Germany; 27 Gerontology Research Center, Tampere University, Tampere, Finland

**Keywords:** nc886, Cardiometabolic disease, Imprinted gene, DNA methylation, vtRNA2-1

## Abstract

Non-coding 886 (*nc886, vtRNA2-1*) is a polymorphically imprinted gene. The methylation status of this locus has been shown to be associated with periconceptional conditions, and both the methylation status and the levels of nc886 RNAs have been shown to associate with later-life health traits. We have previously shown that nc886 RNA levels are associated not only with the methylation status of the locus, but also with a genetic polymorphism upstream from the locus. In this study, we describe the genetic and epigenetic regulators that predict lifelong nc886 RNA levels, as well as their association with cardiometabolic disease (CMD) risk factors and events. We utilised six population cohorts and one CMD cohort comprising 9058 individuals in total. The association of nc886 RNA levels, as predicted by epigenetic and genetic regulators, with CMD phenotypes was analysed using regression models, with a meta-analysis of the results. The meta-analysis showed that individuals with upregulated nc886 RNA levels have higher diastolic blood pressure (β = 0.07, *p* = 0.008), lower HDL levels (β =  − 0.07, *p* = 0.006) and an increased incidence of type 2 diabetes (OR = 1.260, *p* = 0.013). Moreover, CMD patients with upregulated nc886 RNA levels have an increased incidence of stroke (OR = 1.581, *p* = 0.006) and death (OR = 1.290, *p* = 0.046). In conclusion, we show that individuals who are predicted to present elevated nc886 RNA levels have poorer cardiovascular health and are at an elevated risk of complications in secondary prevention. This unique mechanism yields metabolic variation in human populations, constituting a CMD risk factor that cannot be modified through lifestyle choices.

**Supplementary Information:**

The online version contains supplementary material available at 10.1186/s13148-025-01871-7.

## Significance statement

The significance of this research lies in the possibility of uncovering why some individuals might be predisposed to certain CMDs, such as stroke, by factors other than genetics and lifestyle choices. *nc886* presents a unique mechanism contributing to metabolic variation in human populations. The locus is also an example of a regulatory mechanism that is not easily detected in standard genome-wide analyses.

## Introduction

Cardiometabolic diseases (CMDs) are the leading cause of death in the world [1, 2]. While the heritability of some CMDs, such as atherosclerosis, is estimated to be between 50 and 60% within a population [3], large genetic studies have only been able to explain a portion of the heritability [4]. Epigenetic mechanisms, such as DNA methylation, potentially account for the missing heritability that predisposes individuals to CMDs [5, 6]. For example, DNA methylation with low variation within individuals but systemic variation between individuals could mediate metabolic variety in a population and predispose certain subpopulations or individuals to CMDs [7].

DNA methylation has a regulatory function, and it plays an important role in gene expression [8, 9], genomic imprinting [10] and X chromosome inactivation [11]. Genomic imprinting constitutes parent-of-origin-specific expression, caused by silencing of one of the alleles via epigenetic mechanisms, including DNA methylation [12]. For the majority of imprinted genes, imprinting disturbances lead to severe developmental syndromes or embryonic lethality. The role of imprinted genes was originally thought to be relevant only in pregnancy and embryonic growth. However, research has also identified a number of postnatal functions, such as effects on growth, adiposity and glucose metabolism, which play a role in the development of CMD in adulthood [13, 14].

In polymorphically imprinted genes, the methylation status, and thus the gene expression pattern, can vary across individuals. The polymorphic imprinting pattern is commonly regulated by a genetic polymorphism in a sequence that is important for the establishment of the imprint [15]. The only known polymorphically imprinted gene whose methylation is not shown to be associated with genetics in humans is non-coding 886, or *nc886* (official name *vtRNA2-1*) [15–17]. The *nc886* gene is located in a 1.9 kb long differentially methylated region (DMR) of chromosome 5q31.1. Approximately 75% of individuals inherit a methylated allele from their mother and an unmethylated copy from their father, presenting an imprinted *nc886* locus, while roughly 25% of individuals are non-methylated in both alleles [17–19]. We have previously identified a small minority of individuals, 1–6% of the population, who do not fit to this bimodal methylation pattern but rather present an intermediately methylated *nc886* locus, with methylation beta values ranging from 20 to 40%. On an individual level, the methylation pattern of nc886 is stable across the lifespan, and in the majority of tissues [20]. Furthermore, the methylation pattern has been shown to be independent of an individual’s genetics in several publications [17, 19, 21–23], although interestingly, the percentages of different methylation groups have been shown to vary according to the ancestry of the population [24].

The *nc886* locus encodes a 101-nucleotide-long RNA, which is cleaved with low efficiency into two short miRNA-like ncRNAs—nc886-3p and nc886-5p, each approximately 20 nucleotides long [25, 26]. The methylation of the DMR in the *nc886* locus has been shown to associate strongly with the expression of these RNAs, but not directly with any protein-coding genes [17, 22]. Previous work by Park et al. has demonstrated that the methylation of the nc886 region, rather than the methylation of an individual CpG site, leads to changes in the DNA conformation and makes the promoter unreachable to transcription factors [27], supporting the association between DNA methylation level and RNA expression levels. In addition to DNA methylation, we showed that genetic variation within a 100-kb region 92–193 kb downstream from the *nc886* locus, which is hypothesised to contain an enhancer region for the *nc886* gene [28], is associated with nc886-3p and -5p levels in blood [17].

While the *nc886* methylation pattern of an individual is stable across the lifespan, it has been shown to be affected by periconceptional conditions, such as maternal age [17, 18], season of conception [18, 23], maternal nutrition [23] or folic acid supplementation [29], as well as family socioeconomic status [17] and maternal alcohol consumption [18]. *nc886* methylation status has also been associated with childhood BMI [30], adiposity, glucose and cholesterol levels [17]. In addition to the methylation status, the nc886 RNA levels have been associated with allergies [31], asthma [32], infections [33] and inflammation [34]. As the methylation status of the *nc886* locus does not directly affect the expression of any RNAs other than nc886, we hypothesise that nc886 RNA levels mediate the phenotypic effect of the polymorphic imprinting in the region.

*nc886* is one of the only molecules that has been suggested to convey the developmental origins of health and disease (DOHaD) hypothesis, linking individuals’ intrauterine conditions to their health in later life. Unfortunately, studying *nc886* poses numerous challenges. The gene is evolutionally young, and the DMR exists only in primates, with the polymorphic methylation pattern being unique to humans [18, 28]. The methylation pattern is also disturbed by the induction of pluripotency and immortalization, making in vitro studies challenging [20, 27]. Furthermore, nc886 RNA measurements are not readily available in large population cohorts, and the uncertainty regarding the nature of the nc886 RNAs leads to ambiguity as to the correct quantification methods [35].

In order to study the role of this RNA, we first set out to investigate the co-regulation of DNA methylation and genetic variation in blood nc886 levels. We also wanted to study whether the genetic variation affecting nc886 RNA levels is cis-acting by investigating whether the parent of origin of the minor allele associates with the RNA levels. As the expression of nc886 is suppressed by methylation in the maternal allele, a minor allele in the maternal copy should not affect the expression if the SNP is cis-acting. Finally, in order to study the association between nc886 expression and CMD phenotypes, we combined the information on the genetic and epigenetic regulatory elements of nc886 RNA expression to create a proxy for lifelong nc886 expression levels and studied its association with blood pressure, cholesterol and triglyceride levels, as well as indicators of sugar metabolism, CMD events and mortality, in six population-based cohorts and one cardiovascular patient cohort.

## Results

### Study population and demographics

In this study, we analysed the effects of predicted elevated nc886 RNA levels in 9058 individuals from six European population cohorts—the Dietary, Lifestyle and Genetic Determinants of Obesity and Metabolic Syndrome (DILGOM) [36], both collection waves (F4 and FF4) of KORA (Cooperative Health Research in the Region of Augsburg) [37], Northern Finland Birth Cohorts 1966 (NFBC1966) [38, 39], Young Finns Study (YFS) [40]—as well as the North American Bogalusa cohort (the Bogalusa Heart Study) [41, 42] and the German cardiovascular patient cohort LURIC (the LUdwigshafen RIsk and Cardiovascular Health study) [43]. Demographics are presented in Supplementary Table 1. The Young Finns Study was also used to investigate the co-regulation of DNA methylation and genetic variation with the blood nc886 RNA levels. The YFS is a longitudinal study which followed the same individuals from baseline the year of 1980. The measurements used in this study were from 2001, 2007, 2011 and 2018. Notably, the 2018 collection also included the parents of the original participants.

### Epigenetic regulation of nc886 levels

**Fig. 1:**
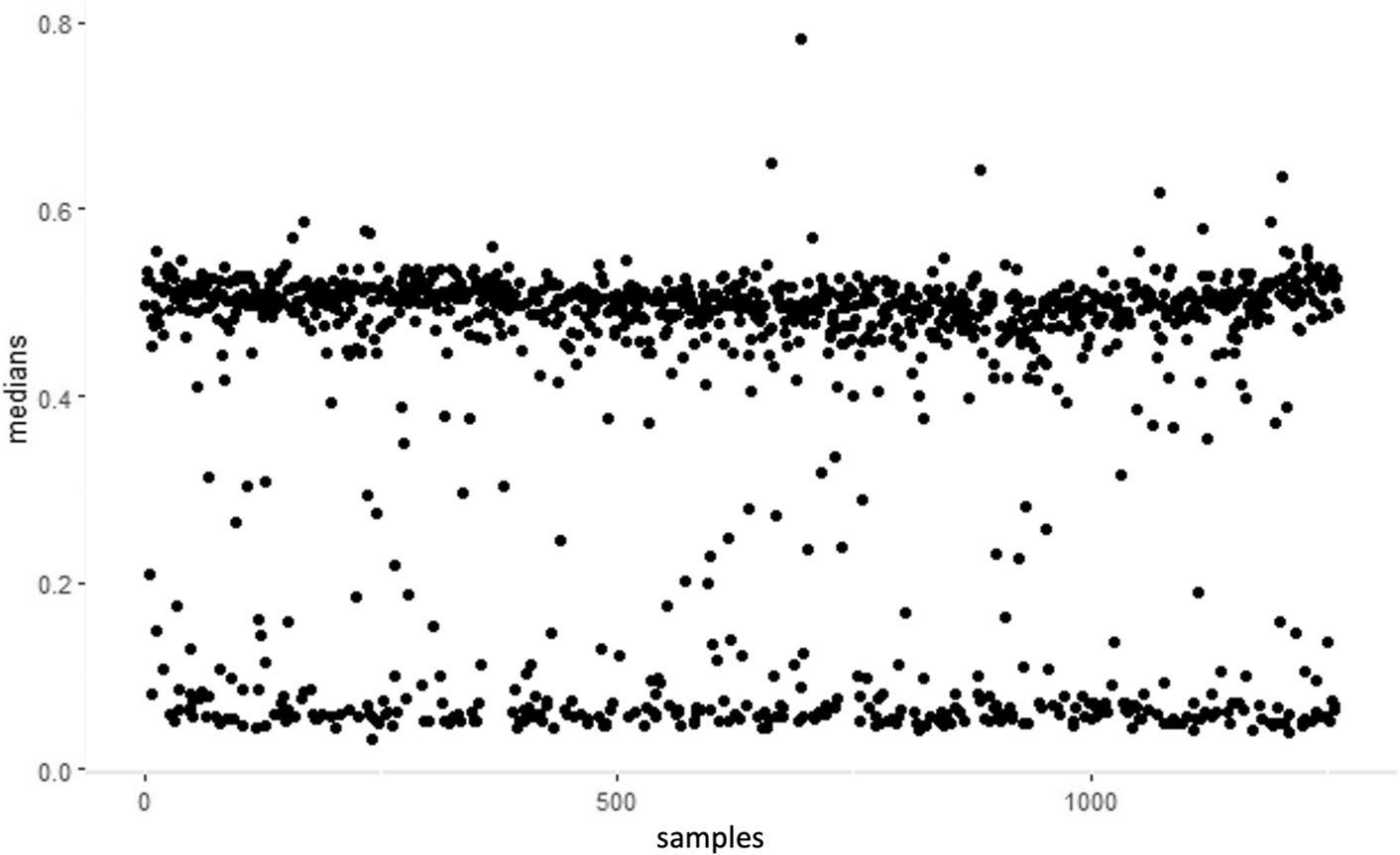
Median methylation of the 11 CpG sites in the *nc886* locus, showing clear bimodal distribution. The samples are from LURIC (*n* = 2183). For all included cohorts, the individuals were clustered into three groups—imprinted (β > 0.4, indicative of monoallelic methylation), intermediately methylated (0.2 < β < 0.4) and non-methylated (β < 0.2, indicative of two unmethylated alleles)

The epigenetic regulation of nc886 RNAs was presented by the median methylation level of 11 CpG sites [17, 18] located on the nc886 DMR, with all of the cohorts included presenting the expected bimodal methylation pattern in the region (Fig. 1). The proportions of imprinted (methylation median > 0.4) and non-methylated (methylation median < 0.2) individuals were uniform across the cohorts included in the study, ranging from 71.6% to 76.5% for imprinted individuals and from 20.9 to 25.0% for non-methylated individuals (Table 1). The largest percentage of intermediately methylated individuals (methylation median 0.2–0.4) was identified in the smallest cohort DILGOM (6.3%), and the corresponding percentage ranged from 2.6% to 4.1% in the other cohorts. Altogether, the observed proportions were well in line with those previously reported for populations of European ancestry [20, 24].

**Table 1:** Proportions of the different genotypes associated with nc886 RNA levels, proportions of the nc886 methylation status groups and the proportions of the combinations of these in the cohorts utilised in the study

		YFS 2007; n = 1497; 30–45 y	DILGOM; n = 504; 25–74 y	LURIC; n = 2183; 17–92 y	KORA F4; n = 1653; 32–81 y	KORA FF4; n = 1771; 38–87 y	Bogalusa; n = 658; 36–57 y	NFBC1966; n = 792; 43–47 y
*nc886 methylation*								
Imprinted		73.20%	72.20%	72.00%	76.50%	75.10%	71.60%	71.80%
Intermethylated		3.20%	6.30%	4.10%	2.60%	3.10%	3.40%	3.20%
Nonmethylated		23.60%	21.40%	23.90%	20.90%	21.70%	24.80%	25.00%
*Genotype*								
T/T		81.60%	85.40%	85.10%	86.80%	86.50%	85.40%	83.10%
T/C		17.40%	14.00%	14.50%	12.70%	12.70%	14.00%	16.10%
C/C		1.00%	0.40%	0.40%	0.50%	0.70%	0.40%	0.80%
*Proxy group*								
Imprinted T/T	59.90%	57.90%	61.30%	66.20%	64.90%	60.90%	59.50%	59,50%
Intermethylated T/T	2.80%	5.90%	3.60%	2.20%	2.60%	2.70%	2.80%	2,80%
Nonmethylated T/T	18.90%	18.40%	20.20%	18.40%	19.00%	21.80%	20.80%	20,80%
Imprinted T/C	12.60%	13.70%	10.40%	9.90%	9.70%	10.30%	12.00%	12,00%
Intermethylated T/C	0.40%	0.40%	0.50%	0.40%	0.50%	0.70%	0.40%	0,40%
Nonmethylated T/C	4.40%	3.00%	3.60%	2.40%	2.50%	3.00%	3.60%	3,60%
Imprinted C/C	0.70%	0.60%	0.30%	0.40%	0.50%	0.40%	0.30%	0,30%
Intermethylated C/C	0%	0%	0%	0%	0%	0%	0%	0%
Nonmethylated C/C	0.30%	0.00%	0.10%	0.10%	0.20%	0.00%	0.60%	0,60%
*Use in proxy*								
Control		59.90%	57.90%	61.30%	66.20%	64.90%	60.90%	59.50%
Elevated nc886 RNA		24.30%	22.00%	24.20%	21.30%	22.20%	25.20%	25.30%
Excluded		15.80%	20.00%	14.50%	12.50%	12.80%	13.70%	15.20%

“Use in proxy” refers to analyses associating the predicted nc886 RNA levels with CMD phenotypes. Intermediately methylated (intermethylated) groups were excluded due to low numbers, and the “Imprinted T/C” group was excluded due to the different effects of a maternally or paternally inherited minor allele, as described in the main body of the text. The total numbers of individuals with available methylation and genotype data, as well as the age ranges, are listed under the cohort names. Other cohort demographics are found in Supplementary Table 1

### Identification of the lead SNP regulating nc886 RNA expression

In order to identify the genetic variation regulating nc886 RNA expression, we utilised the previously reported GWAS/eQTL data demonstrating that genetic variation 100 kb downstream from the nc886 locus is associated with the levels of nc886 RNAs [17]. To identify the lead SNP, or a SNP representing the genetic regulation of the nc886 RNA levels, FUMA [44] was utilised to prioritise the results. The analysis yielded seven lead SNPs for nc886-3p and three for nc886-5p nc886 RNA levels. Association analyses performed between each SNP and the blood levels of nc886-3p RNA showed that the lead SNP rs1799962 (log-additive* p*-value of 2.217*10^60^) had the strongest association with the RNA blood levels (Supplementary Table 2). The most significant SNP for nc886-5p RNA levels (rs58183315) was in almost complete linkage with rs1799962 in the YFS 2011 population (*n* = 1714), with only one individual presenting a different genotype. Thus, rs1799962 was selected to represent the genetic regulation of nc886 RNA levels. Individuals who were heterozygotic for this SNP (T/C) exhibited higher levels of nc886-3p and -5p compared to major allele homozygotes (FC = 2.3 and 1.7, respectively). The effect was even larger in minor allele homozygotes (FC = 3.5 and 2.5, respectively). The prevalence of rs1799962 major allele homozygotes (T/T) was similar in all of the utilised cohorts, ranging from 81.6 to 86.8% (Table 1).

### Combining genetic and epigenetic regulation of *nc886* RNAs

**Fig. 2:**
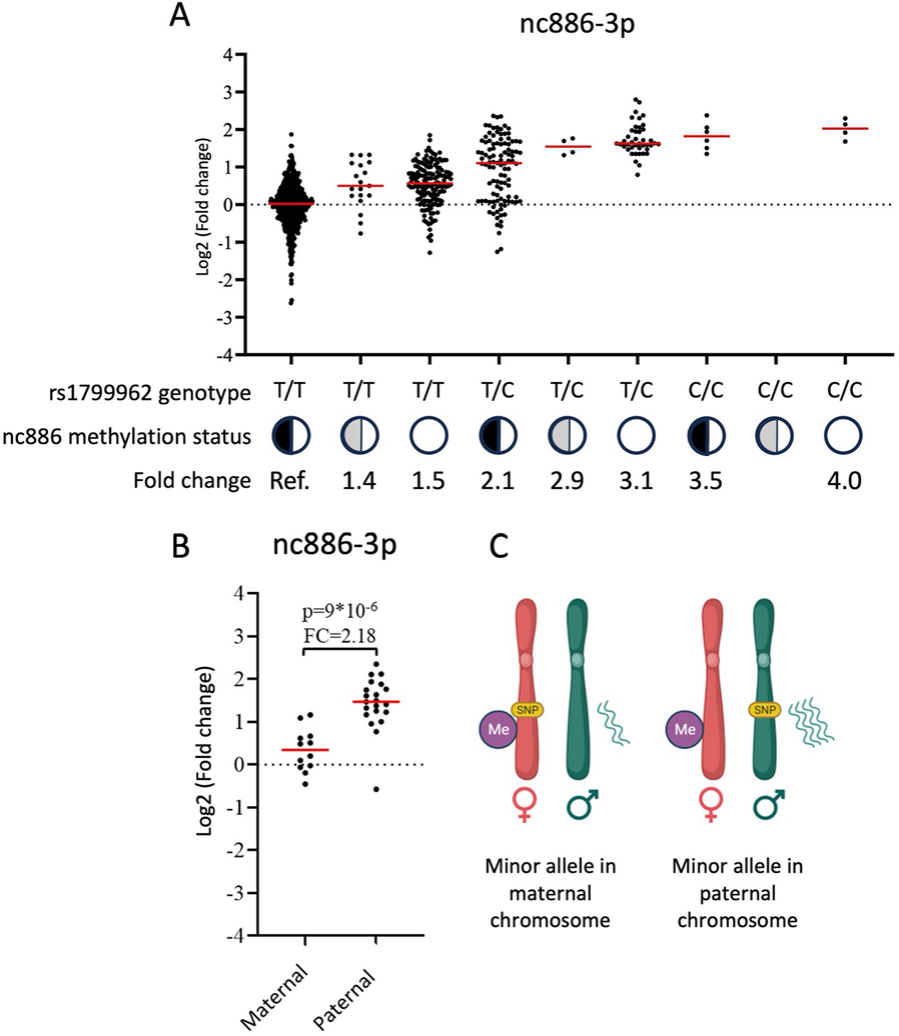
Association of nc886-3p blood RNA levels and epigenetic and genetic regulators using the YFS cohort. **A** Having the *nc886* locus non-methylated in both alleles (white circle) or being a carrier of the minor allele (C allele) of rs1799962 is associated with elevated nc886-3p levels, when compared to imprinted major allele homozygotes (Ref.). In imprinted heterozygotes, the expression of nc886-3p shows a bimodal distribution, whereas the expression in other groups shows a unimodal distribution. The black and white circle represents imprinted individuals, while the grey and white is for intermediately methylated individuals. **B** and **C** A detailed analysis of individuals presenting an imprinted *nc886* locus and a heterozygotic (T/C) rs1799962 genotype reveals that the presence of the minor allele does not have a major effect on the nc886-3p RNA levels when it has been inherited from the mother (located in the silenced maternal allele), while it associates with more than twofold upregulation in the RNA levels when the minor allele is inherited from the father (locates in the transcriptively active paternal allele). This supports the idea that the effect of rs1799962 on nc886-3p levels is cis-acting. The same reference group (ref.) is utilised in both figures A and B

In order to create a proxy for lifelong nc886 RNA levels, we combined the information concerning the two regulatory mechanisms associated with nc886 RNA levels—the genotypes of rs1799962 and the methylation status groups of *nc886*—as shown in Table 1 and Fig. 2. As previously shown, in comparison to individuals with an imprinted *nc886* locus, individuals with a non-methylated or intermediately methylated *nc886* locus present higher blood nc886 RNA levels [17]. In addition, regardless of the methylation status of the *nc886* locus, carriers of the minor allele of rs1799962 (C/T or C/C) present higher RNA levels than major allele homozygotes (T/T) (Fig. 2 for nc886-3p levels and Supplementary Fig. 1 for nc886-5p levels). This combined variable was used to present the stable upregulation of nc886 RNAs. Group frequencies of each cohort are shown in Table 1. Intermediately methylated individuals were removed from further analyses, as their classification from various datasets is ambiguous and their frequency was relatively low. Major allele carriers presenting a non-methylated *nc886* locus and minor allele carriers were grouped as individuals presenting upregulated nc886 levels, while rs1799962 major allele homozygotes with an imprinted *nc886* locus were used as controls (Table 1). Individuals presenting a heterozygotic genotype of rs1799962 and an imprinted *nc886* locus were further removed from the analysis (see section below).

### The effect of the minor allele of rs1799962 on nc886 expression is dependent on the parent of origin

Previous reports have shown that the maternal allele of *nc886* is fully silenced in imprinted individuals [16, 18, 19], and we hypothesised that, if rs1799962 is a cis-acting SNP, there would be a difference in blood nc886 RNA levels with respect to whether the minor allele locates to the allele inherited from the mother or the father. In support of our hypothesis, a bimodal expression pattern of nc886 RNAs was observed in individuals presenting a heterozygotic genotype of rs1799962 and an imprinted *nc886* locus, (Fig. 2A and Supplementary Fig. 1). We then further investigated the subpopulation of these individuals, in whom the parental origin of the minor allele is known (*n* = 33). A significantly higher expression was detected in individuals who have inherited the minor allele from the paternal lineage when compared to individuals who have inherited the minor allele from the maternal lineage (*p* = 9.00*10^–6^, FC = 2.18 for nc886-3p, Fig. 2; and *p* = 3.8*10^–5^, FC = 1.76 for nc886-5p, Supplementary Fig. 1).

Individuals with an imprinted *nc886* locus and a heterozygotic rs1799962 genotype who carry the minor allele in their non-expressive maternal allele have nc886 RNA levels similar to imprinted individuals without the minor allele, which is to say that the minor allele cis to the silenced *nc886* locus has no effect on the total RNA levels. In individuals with an imprinted *nc886* locus and a heterozygotic rs1799962 genotype who carry the minor allele in their paternal copy of the gene, the SNP cis to the active *nc886* locus is associated with increased nc886 RNA levels. As we cannot determine the parent of origin of the minor allele for the majority of the individuals utilised in this analysis, the rs1799962 heterozygotic individuals with an imprinted *nc886* locus were discarded from further analyses.

### Association analyses of nc886 RNA levels and CMD risk factors in individual cohorts

We analysed the association between CMD risk factors, i.e. blood pressure, blood lipids and markers of glucose metabolism, and predicted nc886 RNA levels using a linear regression model. Imprinted major allele homozygotes were used as a control group and they were compared to individuals presenting elevated nc886 RNA levels, as described in previous sections and Table 1. The association analysis was performed in six population cohorts (YFS, DILGOM, KORA F4, KORA FF4, Bogalusa and NFBC1966) and one cardiovascular disease (CVD) patient cohort (LURIC). As the YFS contains phenotypic data from multiple time points, cross-sectional analyses between CVD risk factors and epigenetically and genetically elevated nc886 RNA levels versus controls were performed for each of the time points (the 2001, 2007, 2011 and 2018 follow-ups).

**Fig. 3:**
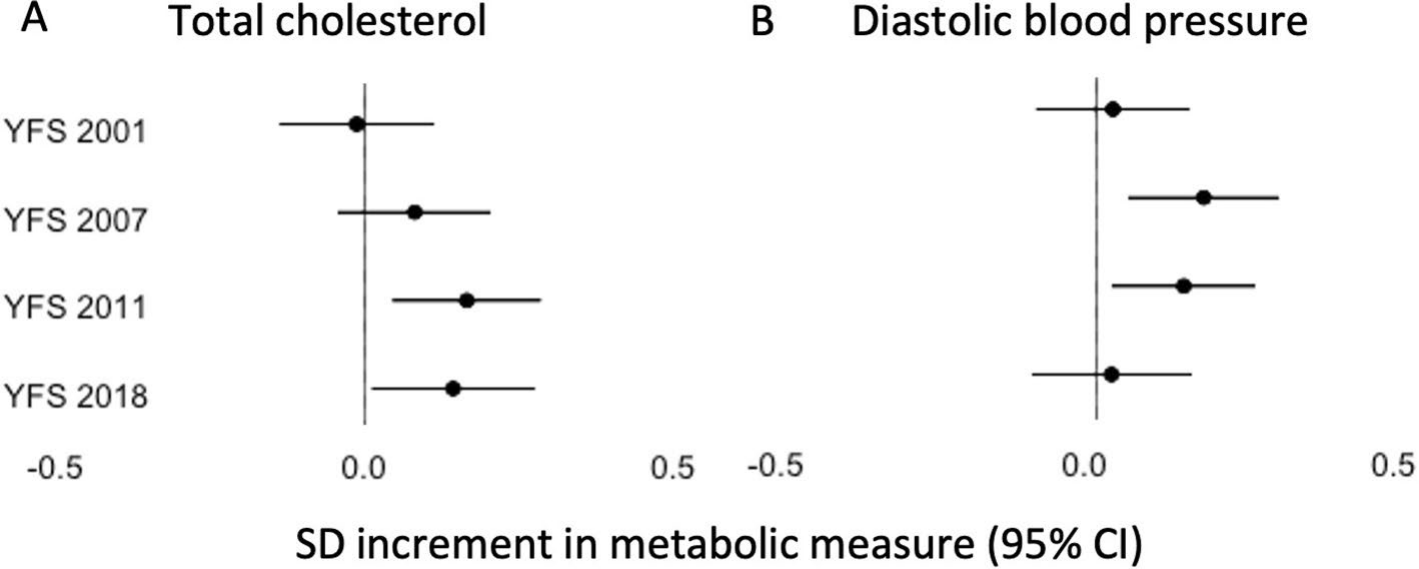
Forest plots of the association of the genetic and epigenetic determinant of *nc886* with **A** total cholesterol in men and **B** diastolic blood pressure in the YFS from 2001, 2007, 2011 and 2018. The control group comprises individuals who are imprinted and major allele homozygotes, and they are compared to individuals with elevated predicted nc886 RNA levels

Regarding blood pressure, we observed that individuals with upregulated nc886 RNA levels had higher diastolic blood pressure than the control group. The association was statistically significant for two time points in the YFS data (Fig. 3B, Supplementary Table 3). In sex-stratified analyses, the association between predicted nc886 RNA levels and diastolic blood pressure was statistically significant in women at one time point in the YFS and in the KORA FF4, as well as in men in the LURIC cohort (Supplementary Table 3). In line with these results, we observed a higher prevalence of hypertension in women with upregulated nc886 RNA levels at one time point in the YFS, as well as in the Bogalusa and NFBC1966 cohorts (Supplementary Table 4). However, an opposite result was observed in KORA, where individuals with upregulated nc886 RNA levels had a lower prevalence of hypertension, in both the KORA F4 and KORA FF4 cohorts, as well as in a sex-stratified analysis (for women in KORA F4 and for men in KORA FF4).

As regards circulating blood lipids, we had data on LDL, HDL, non-HDL and total cholesterol. We observed higher levels of LDL non-HDL and total cholesterol in individuals with upregulated nc886 RNA levels. Statistically significant associations were observed for all three lipids at one time point in the YFS, and in sex-stratified analyses, statistically significant associations were discovered for non-HDL and total cholesterol in women in the Bogalusa study and in men at two time points of the YFS (Fig. 3A) (Supplementary Table 3). HDL cholesterol levels were lower in individuals with upregulated nc886 RNA levels that they were in the controls. The association was statistically significant in LURIC, when analysing the whole dataset, as well as in sex-stratified analyses. An opposite result was observed for men at one time point of the YFS, where individuals with upregulated nc886 RNA levels showed higher levels of HDL cholesterol than did controls (Supplementary Table 3).

Finally, in individuals with upregulated nc886 RNA levels, we observed lower concentrations of plasma glucose one time point in the YFS, as well as in men at one time point in the YFS. Higher concentrations of insulin were observed in individuals with upregulated nc886 RNA levels in NFBC1966 and, in sex-stratified analyses, in women in the DILGOM and NFBC1966 cohorts. We also observed a higher incidence of type 2 diabetes in individuals with upregulated nc886 RNA levels in the KORA FF4, as well as in men in sex-stratified analyses of the KORA F4 (Supplementary Tables 3 and 4).

In LURIC, a CVD patient cohort, our results suggest that individuals with elevated nc886 RNA levels have a significantly higher prevalence of stroke (age range at baseline 17–92 years; prevalence 208 [09.52%], OR = 1.581, 95% CI = 1.138–2.184, *p* = 0.006; Supplementary Table 4). Furthermore, elevated nc886 RNA levels were also associated with a higher incidence of mortality during the 10-year follow-up (age range at baseline 17–92 years; no. of deaths 499 [22.86%], OR = 1.290, 95% CI = 1.003–1.656, *p* = 0.046; Supplementary Table 4).

### Meta-analysis results

**Fig. 4:**
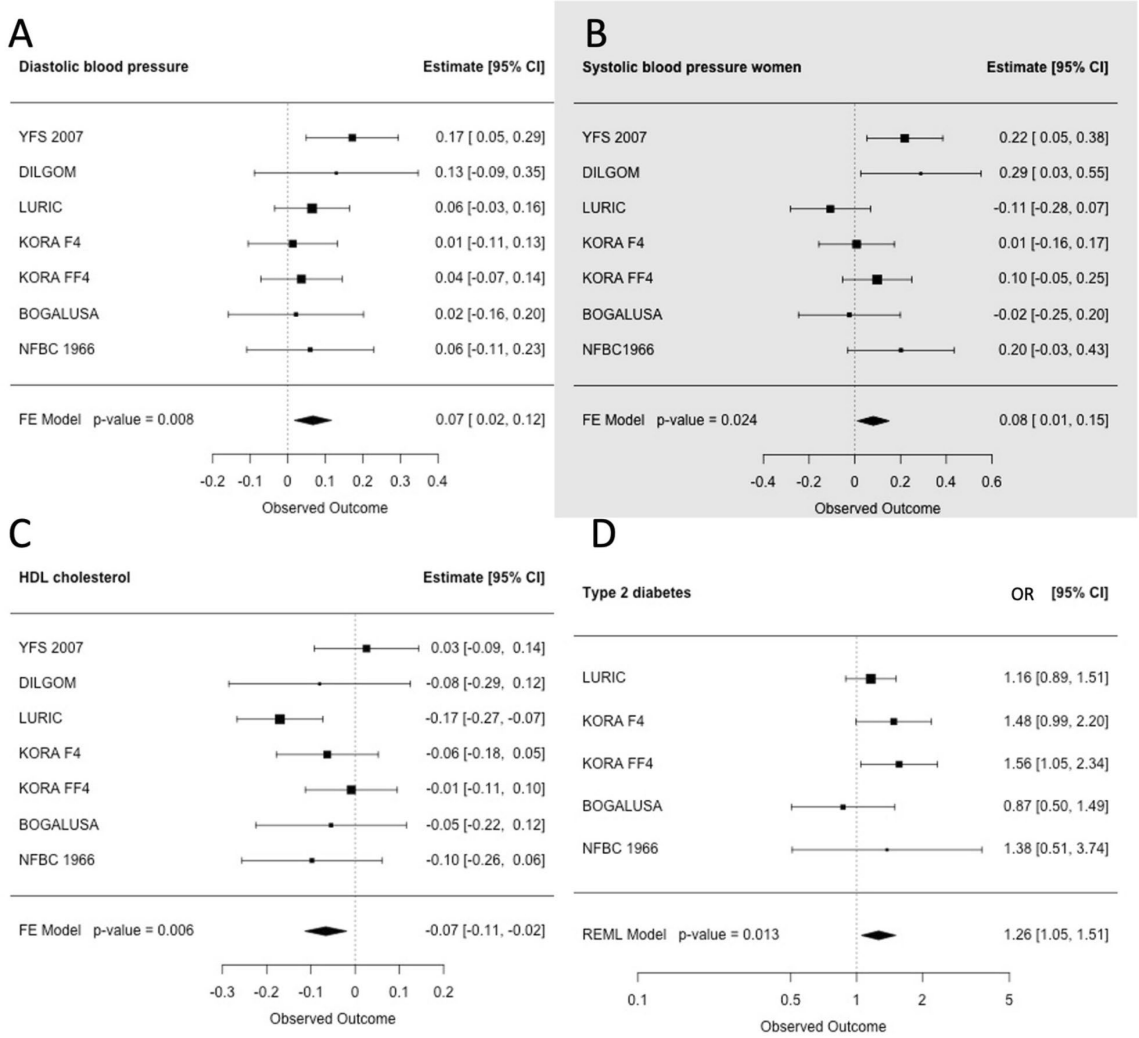
The meta-analysis plots the association of predicted nc886 RNA levels with **A** diastolic blood pressure, **B** systolic bloop pressure in women, **C** HDL cholesterol and **D** type 2 diabetes. Individuals with elevated predicted nc886 RNA levels are compared to imprinted major allele homozygotes. **A** and **B** show diastolic and systolic BP to be significantly upregulated in the meta-analysis results, even though only one/two individual cohorts have a statistically significant association. Meanwhile, HDL, which is often regarded as the ‘good cholesterol’, is significantly downregulated in the meta-analysis shown in **C**, while type 2 diabetes is significantly upregulated, as shown in **D**, which is in line with our hypothesis that elevated nc886 RNA levels contribute to CMD dysregulation

The meta-analysis was performed utilising all available cohorts (only the timepoint 2007 from the YFS, due to the age range of the participants lending itself to the portrayal of metabolic dysfunction that is not yet severe enough to require medication) in order to combine the results regarding the associations in individual cohorts and to study the overall effects of these associations in a systematic manner. In line with the results of individual regression analyses, we show statistically significant upregulation of diastolic blood pressure in individuals with upregulated nc886 RNA levels (*p* = 0.008, *β* = 0.067, 95% CI = 0.017–0.116; Fig. 4A); this result was replicated in the sex-stratified analysis in women (*p* = 0.003, *β* = 0.112, 95% CI = 0.039–0.185) (Supplementary Table 5). In addition, we also observed a higher systolic blood pressure in women (*p* = 0.024, *β* = 0.081, 95% CI = 0.011–0.151) (Supplementary Table 5, Fig. 4B).

We found lower levels of HDL cholesterol in individuals with upregulated nc886 RNA levels compared to the control group (*p* = 0.006,* β* =  − 0.066, 95% CI =  − 0.114–  − 0.019), and the results were again replicated in a sex-stratified analysis in women (*p* = 0.020, *β* =  − 0.087, 95% CI =  − 0.161–  − 0.014) (Supplementary Table 5, Fig. 4C).

Finally, in individuals with predicted elevated nc886 RNA levels, we observed a higher prevalence of type 2 diabetes (OR = 1.260, *p* = 0.013, 95% CI = 1.050–1.510) when compared to controls (Fig. 4D). In the sex-stratified analysis, this was replicated in men (OR = 1.301, *p* = 0.030, 95% CI = 1.025–1.649) (Supplementary Table 5). All meta-analysis plots can be found in Supplementary Fig. 2 for the sexes combined and in Supplementary Figs. 3 and 4 for women and men, respectively.

## Discussion

We have demonstrated herein that the expression of nc886 RNAs in the human population is additively regulated by genetic and DNA methylation variation, with up to fourfold upregulation in individuals in whom both alleles are non-methylated and who are minor allele homozygotes, as opposed to imprinted individuals who are homozygotic carriers of the major allele. By utilising the genetic information of the parents of the study participants, we have shown that the genetic variation is cis-acting and has an effect on RNA expression only when present in an allele that is not silenced by DNA methylation. The prevalence of individuals with genetically and/or epigenetically upregulated nc886 RNA levels is shown to be approximately 30%–35% in all of the populations included in the study, comprising a total of 9058 individuals. Furthermore, our results show, for the first time, that the individuals with genetically and/or epigenetically upregulated nc886 RNA levels demonstrate less optimal cardiovascular health, as they are more likely to have higher blood pressure, diabetes prevalence, higher cholesterol levels, and in a patient cohort of cardiovascular disease patients, are also more likely to have suffered a stroke or died during a follow-up of 10 years.

We and others have previously shown that the expression of nc886 RNAs is strongly regulated by the bimodal methylation status of the *nc886* DMR [17, 22, 45], also demonstrating that blood levels of nc886-3p and -5p are additionally affected by genetic variation 92–193 kb downstream from the gene locus [17]. The area downstream from *nc886* could harbour an enhancer area regulating nc886 expression, as the CTCF-binding site in this region has been shown to interact with another CTCF-binding site flanking the *nc886* DMR [28]. Herein, we identified rs1799962 as the lead SNP representing the genetic variation and showed that it had an additive effect on top of the methylation pattern of the *nc886* DMR on the nc886-3p and -5p RNA levels. The presence of a minor allele is associated with more than 1.7-fold upregulation of nc886 RNA levels, and the effect was even stronger in minor allele homozygotes with more than 2.5-fold upregulation. As the methylation pattern in the *nc886* locus has been shown to be temporally stable and similar in the majority of, although not all, somatic tissues [20, 24], the methylation status of the *nc886* DMR and the genotype of rs1799962 can be regarded as stable regulators of nc886 RNA, which results in its increased expression in individuals with the non-methylated epigenotype and in the presence of the minor allele.

In imprinted individuals, the *nc886* gene locus is regarded to be maternally silenced, based on the methylation patterns of gametes [16, 18–20] and a total of 18 family trios [46, 47]. Park et al*.* have shown that the methylation in the imprinted allele leads to the formation of heterochromatin, making it inaccessible for transcription [27]. In the present study, we further validate the maternal imprinting and cis-acting effect of the SNP rs1799962 by utilising genetic, epigenetic and RNA expression data from the multigenerational YFS cohort. We show that the minor allele of rs1799962 associates with the upregulation of nc886-3p and -5p expression in imprinted individuals only when it is inherited from the father, i.e. it locates in the expressive allele, which is not silenced by methylation.

We have previously reported that individuals with a non-methylated *nc886* epiallele, and thus higher nc886 RNA levels, have higher insulin levels and lower glucose levels during adolescence [17]. Furthermore, we described that boys with a non-methylated epigenotype had elevated concentrations of HDL and non-HDL cholesterol in comparison to imprinted individuals. An analysis with only the methylation status repeated in adults did not show statistically significant metabolic differences, with the exception of non-methylated men in their early 30s having elevated non-HDL cholesterol levels in comparison to imprinted men [17].

In the current study, our association analysis conducted in seven cohorts (with the addition of the longitudinal YFS being analysed here for four separate timepoints) reveals that individuals presenting genetically and/or epigenetically upregulated nc886 RNA levels have elevated non-HDL and total cholesterol in at least one cohort (Supplementary Table 3). In line with previous results, the effect is also seen in men when analysed separately [17]. We also observed statistically significant, albeit small, downregulation of HDL cholesterol in a meta-analysis, which aligns with the direction of metabolic dysfunction. A statistically significant difference can also be seen in insulin and glucose concentrations in at least one cohort, which is also in line with previous results [17]. The meta-analysis of 9058 participants with a diverse range of ages and origins shows an increased risk of type 2 diabetes in participants presenting the more permissive nc866 expression regulation, which is in line with the results from our association studies on the individual cohorts. Moreover, individuals with predicted upregulated nc886 RNA levels have slightly higher diastolic blood pressure. The effect was also significant in the meta-analysis of women alone, with the addition of systolic blood pressure. Intriguingly, in analyses with individual cohorts, we observed significant dysregulation of hypertension in both directions, with the association being negative in the KORA F4 and FF4 and positive for women in three of the other cohorts. In LURIC, which is a cohort of cardiovascular disease patients, we could also see an elevated risk of stoke and death in individual association analyses. The *nc886* gene, DMR methylation or RNA expression has not been previously associated with blood pressure, stroke or death. Epigenome-wide association studies (EWASs) searching for associations between DNA methylation and blood pressure phenotypes [48–50] or stroke [51–53] remain scarce and have focused on individual CpG sites [54]. Furthermore, the linear approach of an EWAS is not suitable for discovering sites with a bimodal methylation pattern and could miss the association completely [24].

Taken together, our results indicate that individuals with a genotype and epigenotype permissive of increased levels of nc886 RNA levels have higher levels of CMD risk factors than do imprinted individuals without the minor allele of rs1799962. As this DNA methylation pattern is thought to be established during the maturation of oocytes [19, 20] and the methylation pattern is temporally stable [17, 23], both the genetic and the epigenetic variation that upregulates nc886 levels may be considered to be predisposing to poorer cardiometabolic health. The role of *nc886* in metabolic health is well in line with the theoretical setting of imprinted genes. It has been theorised that imprinted genes arise from the conflict of interest between the maternal and the paternal genomes. Imprinted genes expressed form the paternal alleles are thought to advance the growth of the developing foetus [55]. In later life, such growth-promoting imprinted genes have been linked with metabolic dysfunction [13, 56].

The methylation pattern of the *nc886* locus has been associated with many physiological phenotypes, but, on a molecular level, it does not associate with the expression levels of any protein-coding genes, only with the non-coding RNAs transcribed from the region [17]. The molecular function of these RNAs is under debate. nc886-102nt has been suggested to function as a pre-miRNA, which is then cleaved into mature miRNAs [26]. Target mRNA analysis and subsequent pathway analysis have indicated targets in insulin signalling, MAPK kinase and chronic myeloid leukaemia pathways [17, 26]. On the other hand, there is building evidence of a direct binding of *nc886* to protein kinase R (PKR) and an inhibition of its activation [25, 35, 57]. Even though PKR is mostly known for its role in viral infection and apoptosis, recent studies have linked it with low-level inflammation, metabolic dysfunction and even the risk of CVDs [58–60]. All suggested mechanisms linking *nc886* function to cardiometabolic diseases remain highly suggestive, and more research is needed to elucidate the mechanisms of how nc886 RNA expression is linked with metabolic health.

The lack of possibility to study the linking mechanism between the regulatory elements of nc886 RNA expression and metabolic dysfunction is one of the major limitations of our study. Furthermore, we have shown the association between these regulatory elements and nc886 RNA expression only in blood. Even though the regulatory elements are stable throughout the majority of somatic tissues [20], the expression levels can be affected by more temporal molecular profiles. The size of the effect of nc886 RNA expression regulation on metabolic traits is small and thus will likely have very little effect on the health of an individual. Furthermore, the results from independent cohorts differ. On the other hand, we were able to demonstrate an association with multiple metabolic traits in seven cohorts with multiple age ranges and genetic backgrounds. The cohorts utilised in the present study also vary in terms of metabolic health, and some of them were medicated for the CVD risk factors studied herein. These traits are also strongly affected by lifestyle choices, and therefore, observing even minor associations across several cohorts is intriguing. Furthermore, as the levels of nc886 RNAs were the highest in minor allele homozygotes and in individuals harbouring the minor allele in the expression-permissive non-methylated chromosome, it would be interesting to study whether the effects on CMD phenotypes are more pronounced in these individuals as opposed to controls. Howbeit, as the prevalence of rs1799962 minor allele carriers is quite low, only up to 18.4% in the studied cohorts, this would require considerably larger population cohorts. In addition, survival analysis was not achieved with the current datasets, and it would be intriguing to see whether the upregulated nc886 RNAs associate with premature mortality.

In conclusion, we describe herein the manner in which two stable regulatory mechanisms, genetic variation and DNA methylation, are associated with the levels of nc886 RNAs, in addition to providing evidence to suggest that the genetic regulation is cis-acting. We show that epigenetically and/or genetically upregulated nc886 RNA levels associate with elevated cholesterol levels and blood pressure, and even with an increased risk of stroke and death in a cohort of cardiovascular disease patients. According to our results, approximately one third of the population belong to this group of individuals with slightly poorer metabolic health throughout their life. The combined regulatory mechanisms of nc886 RNA expression are an example of a non-modifiable risk factor of CMD which is not easily detectable in single-omic studies, representing a unique mechanism which causes metabolic variation in human populations and cannot be modified through lifestyle choices.

## Materials and methods

### Datasets

This study makes use of six population cohorts—DILGOM, KORA F4, KORA FF4, Bogalusa, NFBC1966 and YFS, as well as LURIC, a cohort consisting of patients with cardiovascular diseases (Supplementary Table 1). Combined, these cohorts comprise more than 12,000 individuals, 9058 of whom have methylation data available. From the cohorts, we have utilised methylation data from 11 CpG sites in the *nc886* locus (cg07158503, cg11608150, cg06478886, cg04481923, cg18678645, cg06536614, cg25340688, cg26896946, cg00124993, cg08745965, cg18797653), data on the genotypes of rs1799962, as well as RNA expression (only from the YFS) and phenotype data (plasma levels of LDL, HDL, non-HDL and total cholesterol; systolic and diastolic blood pressure; glucose and insulin concentrations; hypertension; type 2 diabetes; stroke and death, where available).

Bogalusa (The Bogalusa Heart Study) began in 1973 to investigate cardiovascular health in schoolchildren in the town of Bogalusa, Louisiana (approximately 120 km north of New Orleans). Cross-sectional assessments were conducted throughout the 1970s and 1980s, with ongoing cohort follow-up beginning in the 1990s. The current study utilised the data from the core cohort of participants who were examined at least twice during childhood and twice during adulthood, who were born between 1959 and 1979 and whose data were collected during the 2013–2016 visit cycle. A total of 658 participants with both methylation data and genotype data available were included in this study [41, 42].

The Dietary, Lifestyle and Genetic Determinants of Obesity and Metabolic Syndrome (DILGOM) [36] is a subset of The National FINRISK Study (1992–2012), specifically its 2007 survey. FINRISK is a cross-sectional, population-based study aiming to assess the chronic disease risk factors and health behaviours of the Finnish working population aged 25–74 years [61]. The data used for the research were obtained from the THL Biobank (study number THLBB2021_22). DNA methylation and genotype data were available for 504 individuals.

KORA (Cooperative Health Research in the Region of Augsburg) consists of four independent cross-sectional baseline surveys from 1984 to 2001. In total, 17,602 participants were randomly selected from population registries in the study region [37]. The data used in the present study came from two follow-up cycles of the KORA S4 survey—the F4, with data collected in 2006–2008 from 1641 individuals aged 32–81 years, for whom methylation and genetic data are available, and the FF4, with data collected in 2013–2014 from 1873 individuals aged 39–88 years, for whom methylation and genetic data were available. There was an overlap of 988 individuals between the two cycles.

The LUdwigshafen RIsk and Cardiovascular Health study (LURIC) is a cohort of 2183 German individuals aged 17–92 years with and without cardiovascular disease at baseline, for whom methylation and genotype data are available. These individuals underwent coronary angiography between 1997 and 2000 and were subsequently followed up on as regards non-fatal events after five years, as well as on all-cause and cause-specific mortality after 10 years [43].

Northern Finland Birth Cohorts 1966 (NFBC1966) is a population-based birth cohort that invited all pregnant women living in the Oulu and Lapland provinces of Finland with an expected date of delivery in 1966. The cohort originally comprised 96% (*n* = 12,231) of all deliveries that occurred in Northern Finland in 1966. NFBC1966 entailed clinical examinations and/or questionnaire surveys of the children born in 1966 at the ages of 1, 14, 31, 46 and 55 years. DNA methylation and genetic data were measured at the 31-year (*n* = 733) and 46-year (*n* = 716) follow-ups [38]. The current study utilised only the 46-year follow-up.

Young Finns Study (YFS) is a multicentre follow-up study with the aim of determining the impact of lifestyle as well as biological and physiological measures in childhood on the risk of cardiovascular diseases in adulthood. The baseline survey was conducted in 1980, with participants aged 3–18 years. The followed-ups were conducted at three-year intervals until 1992, then again in 2001, 2007, 2011 and finally in 2018–2020 [40]. The DNA methylation data used in the current study are from the 30-year follow-up of 2011, including 1714 participants aged 34–49 years. The phenotypic data utilised in the project are from the 2001, 2007, 2011 and 2018–2020 follow-ups, with the most recent follow-up also including the parents of the original participants.

### DNA methylation

The DNA methylation processing of BOGALUSA [41, 42], DILGOM [36], KORA [17, 62], LURIC [63], NFBC1966 [38] and YFS [17] has been described in detail in the relevant publications concerning the cohorts that are referenced herein. The methylation of genomic DNA was quantified using the Illumina HumanMethylation450 array (DILGOM, KORA F4, Bogalusa) or Illumina EPIC array (NFBC1966, KORA FF4, LURIC) according to the manufacturer’s instructions. Both arrays were used in the YFS 2011 and 2018 follow-ups.

### Clustering

For all datasets utilised, we used median methylation beta values over all participants for 11 CpGs (cg07158503, cg11608150, cg06478886, cg04481923, cg18678645, cg06536614, cg25340688, cg26896946, cg00124993, cg08745965 and cg18797653) that have been shown to display a bimodal DNA methylation pattern in the *nc886* locus, which is indicative of polymorphic imprinting [17, 18]. Three more CpG sites show bimodal expression (cg04515200, cg13581155 and cg11978884) but have been discarded from the analyses due to their tendency towards hypomethylation bias [18]. Based on the mean methylation levels across samples, we clustered the data into three groups – imprinted (β > 0.4, indicative of monoallelic methylation), intermediately methylated (0.2 < β < 0.4), and non-methylated (β < 0.2, indicative of two unmethylated alleles). The cut-off points were defined by the graphic representation of the scatterplots of all mean beta values across each cohort (Fig. 1). We have previously shown that the different normalisation methods between the cohorts do not influence the clustering of the data [17]. As the intermediately methylated individuals do not form a unified group and their number varies between populations, they were removed from further analysis.

### RNA isolation and expression

Whole blood from the YFS participants was collected into PAXgene tubes. RNA from the blood was isolated using appropriate methods. The TaqMan OpenArray MicroRNA Panel, containing nc886-3p and -5p, was used for short non-coding RNA expression profiling, as previously described [17].The nc886 RNA expression data were analysed with the ΔΔ*Cq* method, calculating the nc886-3p and -5p fold changes separately for the ncRNAs in the YFS blood and serum samples, using the median of imprinted individuals as the reference, as previously described [17].

### Genetic analysis

Genotyping for YFS 2011 was performed using a custom-built Illumina Human 670 k BeadChip, measuring 546,677 genotyped SNPs which passed quality control. A genome-wide association study (GWAS)/eQTL analysis on nc886 RNA levels was performed on the YFS data prior to this project, as described in a previous publication [17]. Lead SNP identification was then performed with FUMA, an online platform which is used for analyses such as the annotation and selection of lead SNPs from GWAS data, using default settings [64]. The association between lead SNPs provided by FUMA and whole blood nc886 RNA levels (nc886-3p and nc886-5p) were investigated, and the SNP with the greatest association with the RNAs was selected to represent the genetic contribution to the regulation of nc886 RNAs.

For the parental genome-wide genotyping from the YFS 2018 follow-up, leucocyte DNA was obtained from 1 to 4 ml of EDTA blood samples using a Perkin Elmer Chemagic (CMG-1074) according to the manufacturer’s instructions, with the exception of utilising modified binding buffer 2 with a subset of the samples. The parents of the original participants (*n* = 1996) were genotyped using the Infinium Global Screening Array. Genotypes were called using Illumina's GenCall algorithm. The following filters were used for sample and SNP quality control: sample and SNP call rate < 0.95, cryptic relatedness (pi-hat > 0.2) and SNP Hardy–Weinberg equilibrium test (*p* ≤ 1e-06). Samples with sex discrepancy and excess heterozygosity, as well as genetic outliers detected with multidimensional scaling (MDS), were excluded. Genotype imputation was performed using the Minimac3 and TOPMed r1 reference set on the TOPMed Imputation Server.

Genotyping of the KORA S4/F4 samples was performed on the Affymetrix Axiom Platform. Genotypes were called with the Affymetrix software and annotated to NCBI build 37. Imputation was performed based on the Haplotype Reference Consortium reference panel (Panel (r1.1), April 2016), using minimac3 as an imputation tool (imputation done on Michigan Imputation server) and SHAPEIT v2 as a pre-phasing tool.

In DILGOM, individuals were genotyped using the Illumina Human610-Quad BeadChip [65]; the Bogalusa samples were genotyped using the Illumina Human610 Genotyping BeadChip and HumanCVD BeadChip [66]; the LURIC study employed the Affymetrix Human SNP Array 6.0 [67]; and genotyping of the NFBC cohort was carried out using the Illumina Human CNV370-duo Bead Array during the 31-year follow-up [38].

### Genetic and epigenetic proxy of nc886 RNA levels

The variable proxying the nc886 RNA levels was created by forming all possible combinations of the three genotypes of the identified lead SNP (major allele homozygotes, heterozygotes and minor allele homozygotes) and the previously described *nc886* methylation status groups (Table 1). This was done in order to represent lifelong nc886 RNA levels and to allow us to investigate the added effect of genetics and epigenetics on nc886 expression. Imprinted major allele homozygotes were used as a control group, while other groups excluding intermediately methylated individuals and imprinted heterozygotes were combined to represent individuals with predicted elevated nc886 RNA.

### Association analysis for individual cohorts

The association between predicted nc886 RNA levels and the metabolic phenotypes from the utilised cohorts were analysed using a linear regression model in R. The phenotypes tested were systolic and diastolic pressures, total cholesterol, HDL, non-HDL and LDL cholesterols, as well as glucose and insulin. Information on the analysis and measurements, which were performed using standard laboratory methods for these molecules, are found in the relevant publications of the YFS [40], Bogalusa [41, 42], DILGOM [36], KORA [37], LURIC [43] and NFBC1966 [38] cohorts. All continuous variables were inverse-transform normalised. The model was adjusted for age, sex and fasting. The analyses used imprinted major allele homozygotes as a reference group and compared them to individuals presenting with elevated nc886 RNA levels, excluding intermediately methylated individuals and imprinted heterozygotes (Table 1). Sex-stratified analyses were performed in a similar setting. FDR adjusted p-values were calculated for each cohort separately.

Analyses were also performed between predicted nc886 RNA levels and categorical phenotypes in the utilised cohorts, in a similar setting to the one applied with the continuous variables but without inverse transformation. The phenotypes tested were hypertension and type 2 diabetes, in addition to stroke and death at the 10-year follow-up in LURIC. For hypertension and type 2 diabetes, only the cohorts with more than 10 cases were analysed. The analysis was performed using a simple generalised linear model with the family function set to “binominal” and correcting the model for age and sex.

### Meta-analysis

Meta-analysis of the regression results was performed using the metafor [68] R [69] package, with a fixed-effects model for continuous variables and a random effects model for categorical variables. Included in the meta-analysis were the results from all six cohorts, comprising 9058 individuals of various nationalities. As the YFS is a longitudinal cohort, only the results from 2007 were used, due to the age range of the participants being optimal for portraying metabolic dysfunction that is not yet severe enough to require medication. Both sex-stratified and combined-sex analyses were conducted.

## Ethical approval

DILGOM: the original FINRISK study has been approved by the Coordinating Ethics Committee of the HUS Hospital District; decision numbers 229/E0/2006 and 332/13/03/00/2013. The FINRISK and DILGOM study materials have been transferred to the THL Biobank in accordance with the notification procedure permitted by the Finnish Biobank Act. KORA: The study was approved by the ethics committee of the Bavarian Medical Association and was carried out in accordance with the principles of the Declaration of Helsinki. All study participants signed written informed consent prior to their participation in the study. LURIC: The study plan was approved by the ethics committee of the State Chamber of Physicians of Rhineland-Palatinate. YFS: The study was approved by the 1st ethical committee of the Hospital District of Southwest Finland on 21 September 2010, and by local ethical committees (1st Ethical Committee of the Hospital District of Southwest Finland, Regional Ethics Committee of the Expert Responsibility area of Tampere University Hospital, Helsinki University Hospital Ethical Committee of Medicine, Research Ethics Committee of the Northern Savo Hospital District, and Ethics Committee of the Northern Ostrobothnia Hospital District). The Bogalusa Heart Study was approved by the Tulane University Institutional Review Board, and informed consent was received from all study participants. NFBC: Informed consent was obtained from study participants for the use of their data in the study. Approval for the studies was granted by the ethics committee of the Northern Ostrobothnia Hospital District in Oulu, Finland, in accordance with the Declaration of Helsinki.

## Supplementary Information

Additional file 1.

Additional file 2.

## Data Availability

The datasets utilised here comprise of health-related participant data, and their use is therefore restricted under the regulations on professional secrecy (Act on the Openness of Government Activities, 612/1999) and on sensitive personal data (Personal Data Act, 523/1999, implementing the EU data protection directive 95/46/EC). Due to these legal restrictions, the data from this study cannot be stored in public repositories or otherwise made publicly available. However, data access may be permitted on a case by case basis upon request.
