# Supplementary material for: Regulation of nc886 (vtRNA2-1) RNAs is associated with cardiometabolic risk factors and diseases
Source: Clin Epigenetics. 2025 Apr 29;17:68. doi: 10.1186/s13148-025-01871-7 (PMC12042507; doi:10.1186/s13148-025-01871-7)
Supplement: Supplementary file 1 — Additional file 1. [file 13148_2025_1871_MOESM1_ESM.pptx]

## Slide 1
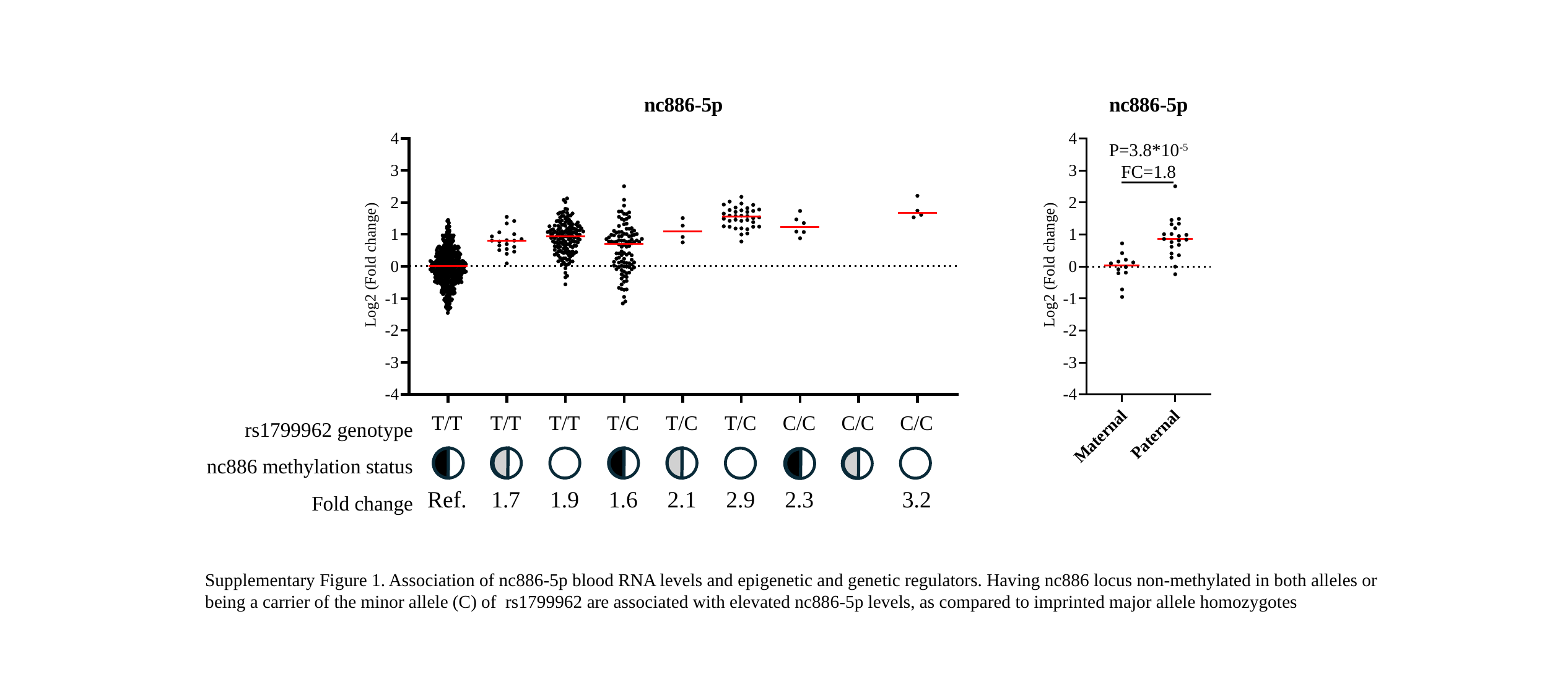

P=3.8*10-5
FC=1.8
rs1799962 genotype
nc886 methylation status
Fold change
| | T/T | T/T | T/T | T/C | T/C | T/C | C/C | C/C | C/C |
| --- | --- | --- | --- | --- | --- | --- | --- | --- | --- |
| | | | | | | | | | |
| | Ref. | 1.7 | 1.9 | 1.6 | 2.1 | 2.9 | 2.3 | | 3.2 |
Supplementary Figure 1. Association of nc886-5p blood RNA levels and epigenetic and genetic regulators. Having nc886 locus non-methylated in both alleles or being a carrier of the minor allele (C) of rs1799962 are associated with elevated nc886-5p levels, as compared to imprinted major allele homozygotes

## Slide 2
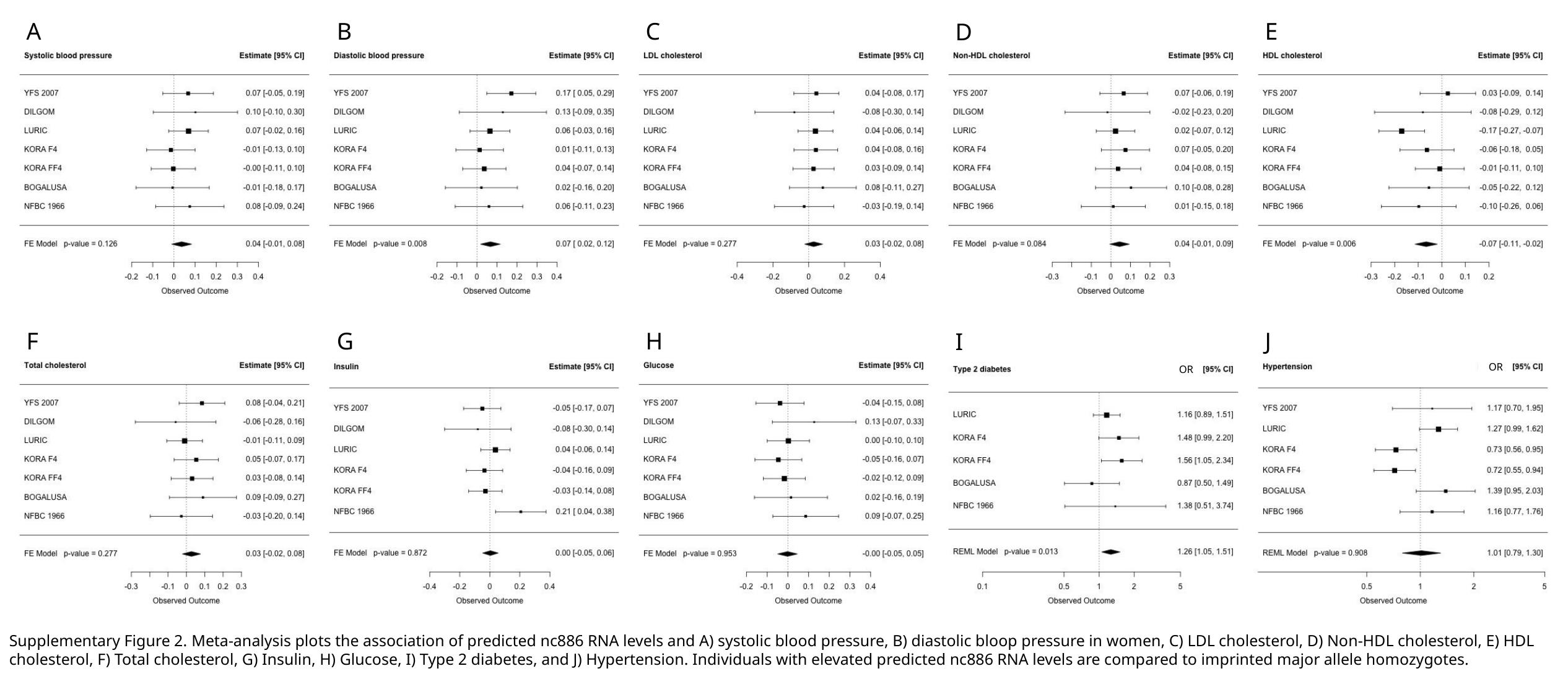

OR
 OR
A
B
C
E
D
F
G
H
J
I
Supplementary Figure 2. Meta-analysis plots the association of predicted nc886 RNA levels and A) systolic blood pressure, B) diastolic bloop pressure in women, C) LDL cholesterol, D) Non-HDL cholesterol, E) HDL cholesterol, F) Total cholesterol, G) Insulin, H) Glucose, I) Type 2 diabetes, and J) Hypertension. Individuals with elevated predicted nc886 RNA levels are compared to imprinted major allele homozygotes.

## Slide 3
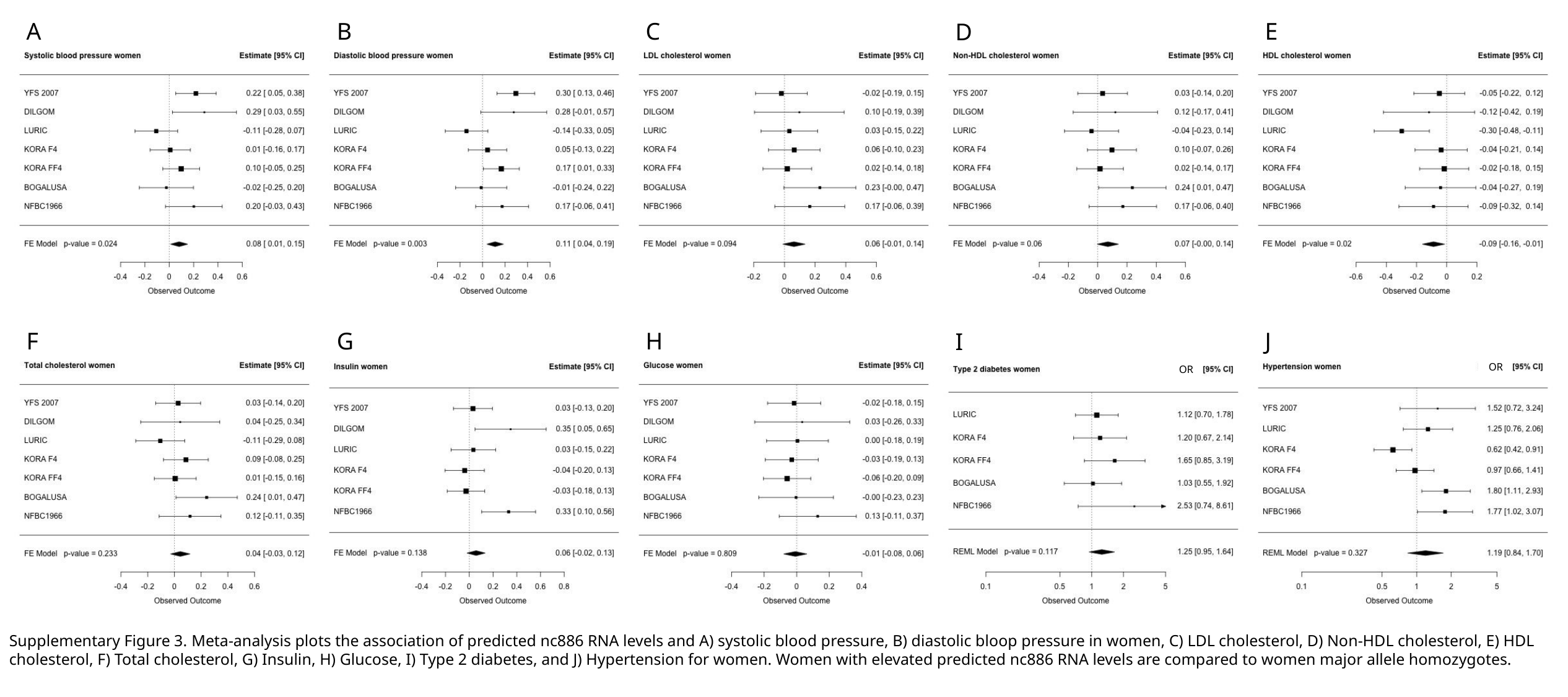

OR
 OR
A
B
C
E
D
F
G
H
J
I
Supplementary Figure 3. Meta-analysis plots the association of predicted nc886 RNA levels and A) systolic blood pressure, B) diastolic bloop pressure in women, C) LDL cholesterol, D) Non-HDL cholesterol, E) HDL cholesterol, F) Total cholesterol, G) Insulin, H) Glucose, I) Type 2 diabetes, and J) Hypertension for women. Women with elevated predicted nc886 RNA levels are compared to women major allele homozygotes.

## Slide 4
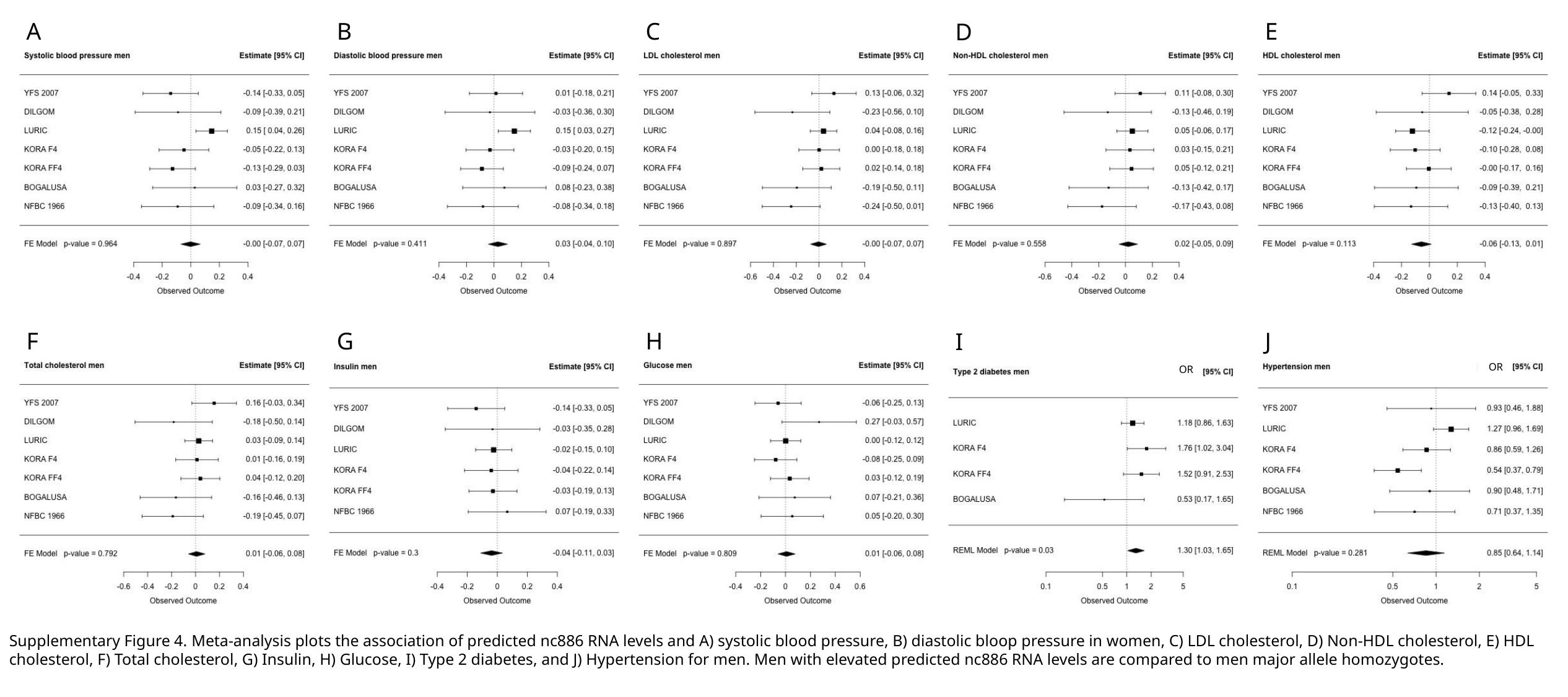

OR
 OR
A
B
C
E
D
F
G
H
J
I
Supplementary Figure 4. Meta-analysis plots the association of predicted nc886 RNA levels and A) systolic blood pressure, B) diastolic bloop pressure in women, C) LDL cholesterol, D) Non-HDL cholesterol, E) HDL cholesterol, F) Total cholesterol, G) Insulin, H) Glucose, I) Type 2 diabetes, and J) Hypertension for men. Men with elevated predicted nc886 RNA levels are compared to men major allele homozygotes.
